# Sleep problems, sleep duration, and use of digital devices among primary school students in Japan

**DOI:** 10.1186/s12889-022-13389-1

**Published:** 2022-05-18

**Authors:** Naoko Sakamoto, Kayoko Kabaya, Meiho Nakayama

**Affiliations:** 1 Faculty of Nursing, Toho University, Omori-nishi, Ohta-ku, Tokyo, 143-0015 Japan; 2 Department of Otolaryngology and Good Sleep Center, Nagoya City University, Kawasumi, Mizuho-cho, Mizuho-ku, Nagoya, 467-8601 Japan

**Keywords:** Weekend sleep duration, Digital device possession, Sleep habits, Public primary school

## Abstract

**Background:**

There is growing concern that screen time and media use in school-age children can negatively affect children’s sleep. These negative effects are explained by three main underlying mechanisms: reduced sleep, time allocated for more media consumption; increased mental, emotional, or psychological stimulation by media content; and the effects of light emitted by digital devices on circadian rhythms and sleep physiology and arousal. In this study, we focused not only on sleep duration, but also on sleep problems. We conducted a large-scale survey to examine the relationship between excessive use of digital devices, Internet addictive behaviour, sleep duration, and sleep problems.

**Methods:**

We conducted a cross-sectional study of children enrolled in 20 public primary schools in Nagoya City, Japan. Children’s parents/guardians completed a questionnaire including the brief sleep questionnaire for Japanese children which is a shortened version of the ‘Children’s Sleep Habits Questionnaire’. Logistic regression analyses were used to identify associations between sleep problems and grade, sex, weekday sleep time, weekend sleep time, ownership of digital devices, frequent checking of digital devices, use of digital devices for more than 4 hours per day, and Internet addiction.

**Results:**

In total, 8172 responses were received (91.6% response rate). After excluding incomplete responses, we analysed complete datasets for 6893 children with a mean age of 9.0 years. When adjusted for sex, grade, sleep duration on weekdays, and sleep duration on weekends, failure to control (odds ratio [OR] = 1.48; 95% confidence interval [CI]: 1.29–1.70; *p* < .001), more use than intended (OR = 1.27; 95% CI: 1.12–1.44; *p* < .001), and use to escape a dysphoric mood (OR = 1.30; 95% CI: 1.03–1.64; *p* = .027) were associated with children’s sleep problems. A shorter weekday and a longer weekend sleep duration indicated a higher likelihood of sleep problems.

**Conclusions:**

After adjusting for sleep duration, a relationship was found between the three Internet addictive behaviours and sleep problems, but not ownership of digital devices. Parents and teachers may need to address screen media-related sleep problems in children, as these problems may be influenced by psychological factors.

## Background

Although the health effects of digital media exposure on children, including sleep, have been emphasised in the past, the coronavirus pandemic has resulted in an increase in media exposure among children [1, 2]. Screen time and screen-based media use in school-aged children and adolescents have been of concern since before the pandemic [3, 4]. A systematic review found that 90% of the 67 studies reviewed reported negative effects regarding screen time, including at least one negative sleep outcome—primarily delayed timing (bedtimes) and shortened duration [5]. These negative effects are explained by three main underlying mechanisms: reduced sleep, time allocated for more media consumption; increased mental, emotional, or psychological stimulation by media content; and the effects of light emitted by digital devices on circadian rhythms and sleep physiology and arousal [6]. While there is general agreement on the association between screen time and poor sleep outcomes, most studies concerning screen-based media use and sleep in children and adolescents have been observational, making it impossible to confirm a causal relationship between the two.

Information and communication technology (ICT) has become prevalent in the lives of children. ICT is being used as an innovative tool in variety of ways in education, in play, especially games, and in every day communication. Problematic behaviours related to the Internet such as ‘excessive Internet use’, ‘Internet addiction’, and ‘pathological Internet use’ due to inappropriate ICT use [7–13] have become apparent. Concerns about the potential harms of Internet use are rapidly growing, especially considering that gaming disorders have been included as a formal disorder in the International Classification of Diseases 11th Revision [14] and as a disorder requiring further study in the Diagnostic and Statistical Manual of Mental Disorders, Fifth Edition [15]. Epidemiological studies on Internet addiction prevalence have reported large variations, from 0.5–40.0% [16]. The prevalence of Internet addiction may vary depending on study design, assessment scales, sampling, and the extent of digital device penetration. A large study of more than 10,000 adolescents in 11 EU countries showed a prevalence of 4.4% in 2012 [17]. Asian countries have a high prevalence of Internet addiction among young people, with reported rates of 10.4% in China [18], 10.7% in South Korea [19], and 17–26.8% in Hong Kong [20]. Addiction is a serious health problem in itself, but it can also interfere with children’s sleep and thus with their physical and mental development [21–24]. In Japan, a 2018 survey of junior high school students (aged 12–15 years) reported that 4.3% were addicted to the Internet [13].

Sleep plays an important role in children’s health and development, such as brain development and plasticity [25], and disrupted sleep is associated with decline in cognitive and behavioural functioning and poor academic performance [26–31]. Previous studies have shown that the use of screen-based media shortens sleep time, disrupts sleep onset, and affects circadian rhythms in children, and that many children are Internet-dependent. Furthermore, in children and adolescents, a temporal relationship has been shown in which insomnia in the early and middle stages predicts Internet addiction and circadian rhythm disorders [21]. Therefore, it is vital to understand the association between Internet use and sleep problems in children in the current digital world. However, few studies have investigated the impact of Internet-dependency on sleep problems in children, which is also a serious issue in Japan. We examined the relationship between excessive Internet use and sleep problems in Japanese primary school students using a large-scale survey.

## Methods

### Participants and study design

This was a cross-sectional study of students enrolled in 20 public primary schools in Nagoya City, one of the largest cities in Japan with a population of 2,327,557. It was conducted in October 2019 [32]. In the same year, Nagoya’s 2019 monthly average per working household was 546,611 yen (4800 USD), while that of Japan in 2019 was 586,149 yen (5100 USD) [33]. The total number of primary school students in Nagoya City that year was 112,106 (57,643 boys and 54,463 girls) [34]. We used a stratified random sampling method to extract 20 schools from the city across 16 school districts: one school each from 12 school districts and two schools each from four districts with a relatively large population. Self-administered, anonymous questionnaires and documents explaining the research purpose and ethical considerations were distributed at the schools. Parents/guardians voluntarily agreed to participate in the study and returned the questionnaires upon completion in anonymous envelops. They could also choose to not respond. We received 8172 responses (response rate = 91.6%). Excluding records with even one missing value, 6893 records were analysed.

Ethical approval was granted by the Nagoya City University Medical Research Ethics Review Committee (no. 60-19-0108), and approval from the Nagoya City Board of Education was obtained. However, because we could not obtain approval from the Nagoya City Board of Education to collect information on the sociodemographic status of the parents/guardians, we did not collect such information in this study.

### Questionnaire

Sleep parameters are generally divided into four categories: duration, quality, sleep architecture and brain activity patterns, and schedule or circadian aspects [35]. In this study, bedtime was classified as schedule information, weekday and weekend sleep duration as duration information, and the brief sleep questionnaire for Japanese children was classified as quality information. For a large-scale epidemiological study, we did not collect sleep structure and brain activity pattern information. The brief sleep questionnaire for Japanese children consists of 19 question items out of 52 in the Japanese version of the Children’s Sleep Habits Questionnaire (CSHQ-J) [36, 37]. The Children’s Sleep Habits Questionnaire (CSHQ) has been translated and is being used in various countries to assess sleep patterns and sleep problems in children [38–41]. The Cronbach’s alpha of the brief sleep questionnaire is 0.65, indicating acceptable internal consistency [42]. Okada confirmed that the cumulative score of the brief questionnaire (cut-off score = 24) was effective as a discriminator by comparing children with sleep problems (CSHQ total score ≥ 41) with children without sleep problems (CSHQ total score < 41) using the CSHQ cut-off value [36]. Using the cut-off score of the original CSHQ as a threshold, sensitivity and specificity were examined using receiver operating characteristic curve analysis, and the area under the curve was 0.89 (0.86–0.91), sensitivity was 0.83, specificity was 0.78, and the cut-off score was 24 [36]. The cumulative score of the brief questionnaire correlated with the cumulative score of CSHQ was *r* = 0.81. It was also significantly correlated with the subscale score (*r* = 0.29–0.65) [36].

We also asked the participants three questions about the use of digital devices: (1) Does your child have a mobile phone, smartphone, tablet, or personal computer? (2) Does your child seem to be constantly checking their digital devices? (3) Does your child use digital devices for more than 4 hours a day?

We used a Japanese questionnaire on Internet addiction for junior and senior high school students [43], which was developed based on the Young Diagnostic Questionnaire for Internet Addiction (YDQ) [44]. It was converted into a format for parents/guardians to answer. The YDQ consists of eight questions, and if five or more items are met, it is suspected that the person is addicted to the Internet: (1) Do you think your child is preoccupied with the Internet? (thinks about previous on-line activity or anticipates their next on-line session)? (2) Do you think your child needs to use the Internet more often to achieve satisfaction? (3) Do you think your child repeatedly makes unsuccessful efforts to control, cut back, or stop Internet use? (4) Do you think your child feels restless, moody, depressed, or irritable when attempting to cut down or stop Internet use? (5) Do you think your child stays on-line longer than originally intended? (6) Do you think your child jeopardised or risked the loss of a significant relationship, educational, or extracurricular activity because of the Internet? (7) Do you think your child lied to family members or teachers to conceal the extent of their Internet involvement? (8) Do you think your child uses the Internet as a way of escaping from problems or for relieving a dysphoric mood?

### Statistical analysis

To describe the data, we analysed the means and standard deviations of weekday and weekend bedtime, weekday and weekend sleep duration, and the cumulative score of the brief sleep questionnaire for Japanese children by grade/sex. The Jonckheere–Terpstra test was performed to examine grade trends in the quantitative data. Additionally, the Cochrane–Armitage trend test was conducted to examine grade trends in the percentage of answers to the three questions regarding digital device use and the YDQ. In the logistic regression analyses, the dependent variable was whether the cumulative score of the brief sleep questionnaire for Japanese children was 24 or more. The independent variables were grade, sex, sleep duration on weekdays, sleep duration on weekends, digital device possession, checking digital devices frequently, digital device use for more than 4 hours per day, and the YDQ. We built a subset model using backward stepwise logistic regression analyses (likelihood ratio) to clarify the items related to Internet addiction that contribute to children’s sleep while adjusting for grade, sex, sleep duration on weekdays, sleep duration on weekends, and digital device possession.

Significance was set to 5% in all tests. We used SPSS Version 26 (IBM SPSS Statistics for Windows, Version 26.0. Armonk, NY: IBM Corp.) and JMP® Pro 15.2 (SAS Institute Inc., Cary, NC, USA) for all analyses.

## Results

Complete datasets for 6893 children (grade 1, *n* = 1189; grade 2, *n* = 1165; grade 3, *n* = 1200; grade 4, *n* = 1107; grade 5, *n* = 1070; and grade 6, *n* = 1162) were available for analysis, after excluding incomplete questionnaires. The age range was 6–12, with a mean age of 9.0 ± 1.8. The percentages for boys in each grade, in order from first to sixth grade, were 50.5, 48.6, 50.3, 51.7, 51.7, and 48.4, respectively, and 50.2 overall. Table 1 presents children’s weekday and weekend bedtime, sleep duration, and total score for the brief sleep questionnaire by sex and by grade. For both weekdays and weekends, bedtime progressively occurred at later times and sleep duration decreased with advancing age. On average, children went to bed at 21:34 ± 0:44 for boys and 21:38 ± 0:46 for girls on weekdays, and 22:00 ± 0:52 for boys and 22:04 ± 0:52 for girls on weekends. The mean sleep duration was 8.9 ± 0.8 hours for boys and 8.8 ± 0.8 hours for girls on weekdays, and 9.1 ± 0.9 hours for boys and 9.3 ± 0.9 hours for girls on weekends. As for the cumulative score of the brief sleep questionnaire, the mean score was 24.6 for boys and 24.8 for girls.

**Table 1:** Bedtime, sleep duration, and cumulative score for brief sleep questionnaire for Japanese children by sex/grade

	Sex	Grade 1		Grade 2		Grade 3		Grade 4		Grade 5		Grade 6		*p*-trend^*^
		Mean	*SD*	Mean	*SD*	Mean	*SD*	Mean	*SD*	Mean	*SD*	Mean	*SD*	
Weekday bedtime	boy	21:09	0:38	21:20	0:40	21:27	0:38	21:36	0:39	21:53	0:41	22:06	0:45	< 0.001
	girl	21:11	0:38	21:22	0:39	21:26	0:40	21:42	0:41	22:00	0:43	22:10	0:46	< 0.001
Weekend bedtime	boy	21:33	0:48	21:42	0:47	21:54	0:46	22:04	0:49	22:19	0:49	22:30	0:54	< 0.001
	girl	21:36	0:46	21:48	0:47	21:53	0:47	22:10	0:52	22:22	0:50	22:36	0:48	< 0.001
Weekday sleep duration, hours	boy	9.3	0.7	9.1	0.7	9.0	0.7	8.8	0.7	8.6	0.7	8.4	0.8	< 0.001
	girl	9.3	0.7	9.1	0.7	9.0	0.7	8.8	0.7	8.4	0.7	8.3	0.8	< 0.001
Weekend sleep duration, hours	boy	9.5	0.8	9.3	0.8	9.1	0.8	8.9	0.9	8.9	0.9	8.8	0.9	< 0.001
	girl	9.5	0.8	9.5	0.9	9.3	0.8	9.2	0.8	9.1	0.9	9.0	1.0	< 0.001
Cumulative score of the brief sleep questionnaire for Japanese children	boy	24.9	3.2	25.0	3.3	24.4	3.2	24.7	3.5	24.5	3.3	24.4	3.2	< 0.001
	girl	25.0	3.3	25.0	3.4	24.9	3.2	24.7	3.2	24.6	3.1	24.6	3.5	< 0.001

*SD* is an abbreviation for Standard Deviation

^*^The Jonckheere–Terpstra test was performed to examine grade trends

Among both boys and girls, with an increase in grade, bedtime tended to be delayed on weekdays (boys: TJT = 3,344,346.00, z = 26.07, *p* < .001; girls: TJT = 3,340,581.50, z = 27.18, *p* < .001) and on weekends (boys: TJT = 3,234,237.50, z = 22.73, *p* < .001; girls: TJT = 3,233,242.00, z = 23.87, *p* < .001), sleep duration was shortened on weekdays (boys: TJT = 1,689,776.50, z = − 24.81, *p* < .001; girls: TJT = 1,598,541.50, z = − 26.84, *p* < .001) and weekends (boys: TJT = 1,951,481.50, z = − 16.68, *p* < .001; girls: TJT = 2,073,135.50, z = − 12.08, *p* < .001), and cumulative scores on the brief sleep questionnaire tended to decrease (boys: TJT = 2,353,063.00, z = − 4.10, *p* < .001; girls: TJT = 2,338,133.00, z = − 3.64, *p* < .001).

Table 2 presents the proportion of children with a cumulative score ≥ 24 on the brief sleep questionnaire and the proportion of answers for digital device use questions and YDQ by sex and grade. The mean proportion of students with scores ≥24 was 57.8% (95% confidence interval [CI]: 56.1–59.4%) in boys and 61.1% (CI: 59.5–62.7%) in girls. The mean proportion of having a mobile phone, smartphone, tablet, or personal computer among boys was 41.0% (CI: 39.3–42.6%) and among girls was 46.2% (CI: 44.6–47.9%). The mean proportion for checking digital devices constantly was 18.2% (CI: 16.9–19.5%) for boys and 19.1% (CI: 17.8–20.4%) for girls, and digital device use for more than 4 hours a day was 18.2% (CI: 16.9–19.5%) for boys and 9.2% (CI: 8.3–10.2%) for girls.

**Table 2:** Sleep problems, digital devices and YDQ by sex/grade

	Sex	Grade 1 *n* = 1310		Grade 2 *n* = 1252		Grade 3 *n* = 1278		Grade 4 *n* = 1199		Grade 5 *n* = 1162		Grade 6 *n* = 1282		*p*-trend*
		n	%	n	%	n	%	n	%	n	%	n	%	
Cumulative score of the brief sleep questionnaire for Japanese children ≥24	boy	370	61.7	362	64	331	54.8	327	57.2	299	54.1	308	54.8	< 0.001
	girl	373	63.3	377	62.9	374	62.8	321	60	315	60.9	340	56.7	0.01
Having their own digital device (e.g. smartphone, tablet, PC)	boy	136	22.7	160	28.3	257	42.5	270	47.2	275	49.7	318	56.6	< 0.001
	girl	136	23.1	186	31.1	271	45.5	281	52.5	328	63.4	387	64.5	< 0.001
Checking digital devices frequently	boy	50	8.3	65	11.5	101	16.7	106	18.5	133	24.1	174	31	< 0.001
	girl	48	8.1	52	8.7	85	14.3	87	16.3	155	30	228	38	< 0.001
Using digital devices for more than 4 hours a day	boy	34	5.7	46	8.1	59	9.8	72	12.6	93	16.8	127	22.6	< 0.001
	girl	23	3.9	35	5.8	38	6.4	42	7.9	72	13.9	107	17.8	< 0.001
Five or more checks on the Young Diagnostic Questionnaire for Internet Addiction	boy	89	14.8	85	15	116	19.2	137	24	139	25.1	165	29.4	< 0.001
	girl	47	8	61	10.2	69	11.6	67	12.5	86	16.6	117	19.5	< 0.001
Do you think your child is preoccupied with the Internet?	boy	324	54	309	54.6	342	56.6	333	58.2	330	59.7	363	64.6	< 0.001
	girl	237	40.2	262	43.7	281	47.1	246	46	281	54.4	340	56.7	< 0.001
Do you think your child needs to use the Internet with increasing amounts of time in order to achieve satisfaction?	boy	245	40.8	233	41.2	288	47.7	286	50	294	53.2	342	60.9	< 0.001
	girl	192	32.6	220	36.7	224	37.6	239	44.7	276	53.4	339	56.5	< 0.001
Do you think your child repeatedly made unsuccessful efforts to control, cut back, or stop Internet use?	boy	180	30	168	29.7	191	31.6	213	37.2	217	39.2	240	42.7	< 0.001
	girl	106	18	128	21.4	141	23.7	132	24.7	153	29.6	198	33	< 0.001
Do you think your child feels restless, moody, depressed, or irritable when attempting to cut down or stop Internet use?	boy	170	28.3	164	29	191	31.6	179	31.3	199	36	224	39.9	< 0.001
	girl	89	15.1	107	17.9	118	19.8	98	18.3	115	22.2	142	23.7	< 0.001
Do you think your child stays on-line longer than originally intended?	boy	198	33	209	36.9	214	35.4	260	45.5	270	48.8	303	53.9	< 0.001
	girl	133	22.6	176	29.4	172	28.9	181	33.8	198	38.3	261	43.5	0.001
Do you think your child jeopardized or risked the loss of a significant relationship, educational, or extracurricular activities because of the Internet?	boy	10	1.7	12	2.1	22	3.6	25	4.4	20	3.6	31	5.5	< 0.001
	girl	2	0.3	7	1.2	11	1.8	13	2.4	14	2.7	32	5.3	< 0.001
Do you think your child lied to family members, a therapist, or others to conceal the extent of involvement with the Internet?	boy	46	7.7	50	8.8	61	10.1	75	13.1	82	14.8	113	20.1	< 0.001
	girl	16	2.7	37	6.2	46	7.7	50	9.3	48	9.3	61	10.2	< 0.001
Do you think your child uses the Internet as a way of escaping from problems or relieving a dysphoric mood?	boy	22	3.7	20	3.5	42	7	43	7.5	48	8.7	57	10.1	< 0.001
	girl	16	2.7	11	1.8	28	4.7	25	4.7	43	8.3	43	7.2	< 0.001

*Cochrane Armitage trend test was done to examine grade trends

For both boys and girls, with an increase in grade, the mean proportions of having a mobile phone, smartphone, tablet, or personal computer (both *p* < .001), checking digital devices constantly (both *p* < .001), and digital device use for more than 4 hours a day (both *p* < .001) increased. Conversely, as grade increased, the mean proportions of children with cumulative score ≥ 24 on the brief sleep questionnaire decreased for both boys and girls (boys, *p* < .001; girls, *p* = .01).

The mean proportion of children who scored five or more on the YDQ was 21.1% (CI: 19.8–22.5%) in boys and 13.0% (CI: 11.9–14.2%) in girls. The bottom half of Table 2 shows the proportion of students whose parents/guardians answered ‘yes’ to the eight questions of the YDQ. For both boys and girls, the proportion of respondents to all questions increased with an increase in grade (*p*-trend < .001 or = .001).

We used logistic regression analyses to investigate the relationship among screen-based media usage behaviour, the Internet and Japanese children with a cumulative score ≥ 24 on the brief sleep questionnaire. Table 3 presents the unadjusted odds ratios (ORs) and the results of logistic regression analyses for having a cumulative score ≥ 24 on the brief sleep questionnaire, with the model including all variables except bedtime-weekdays and bedtime-weekends, and the model after backward elimination (likelihood ratio). When adjusted for sex, grade, sleep duration-weekdays, and sleep duration-weekends in the all-variable model, ‘have own digital device’; ‘feel restless, moody, depressed, or irritable when attempting to cut down or stop Internet use’; and ‘stay on-line longer than originally intended’ had a significant effect on children’s cumulative score of the brief sleep questionnaire. In the elimination model, when adjusting for sex, grade, sleep duration-weekdays, and sleep duration-weekends, ‘feel restless, moody, depressed, or irritable when attempting to cut down or stop Internet use’; ‘stay on-line longer than originally intended’; and ‘use the Internet as a way of escaping from problems or for relieving a dysphoric mood’ had a significant effect on children’s cumulative score of the brief sleep questionnaire. In both models of logistic analysis, sex, weekday sleep duration, and weekend sleep duration were statistically significant, and the respective OR values in both models were nearly the same. Being a girl rather than a boy, sleeping less on weekdays, and sleeping more on weekends were associated with having a cumulative score ≥ 24 on the brief sleep questionnaire.

**Table 3:** Odds ratios from logistic regression for having a cumulative score of 24 or more

	Unadjusted				Model of all variables				Model after Backward Elimination			
	OR	95% CI for OR		*p*	OR	95% CI for OR		*p*	OR	95% CI for OR		*p*
		Lower	Upper			Lower	Upper			Lower	Upper	
Grade												
1	1	reference			1	reference			1	reference		
2	1.04	0.88	1.23	0.635	0.99	0.84	1.17	0.907	0.99	0.84	1.18	0.927
3	0.85	0.73	1.01	0.062	0.79	0.66	0.93	0.005	0.79	0.67	0.93	0.006
4	0.85	0.72	1	0.053	0.74	0.62	0.89	0.001	0.75	0.63	0.89	0.001
5	0.81	0.68	0.96	0.013	0.66	0.55	0.79	< 0.001	0.66	0.55	0.79	< 0.001
6	0.76	0.64	0.89	0.001	0.58	0.48	0.7	< 0.001	0.58	0.48	0.69	< 0.001
Sex												
boy	1	reference			1	reference			1	reference		
girl	1.15	1.04	1.27	0.005	1.21	1.1	1.34	< 0.001	1.21	1.09	1.33	< 0.001
Weekday sleep duration, hours	0.91	0.86	0.97	0.004	0.82	0.75	0.88	< 0.001	0.82	0.76	0.89	< 0.001
Weekend sleep duration, hours	1.07	1.01	1.13	0.023	1.17	1.09	1.25	< 0.001	1.16	1.09	1.25	< 0.001
Have their own digital device												
No	1	reference			1	reference			1	reference		
Yes	1.13	1.03	1.25	0.012	1.13	1.01	1.27	0.034	1.11	1	1.23	0.058
Constantly checking his/her digital device												
No	1	reference			1	reference						
Yes	1.19	1.05	1.35	0.007	0.91	0.78	1.07	0.248				
Using digital devices for more than 4 hours a day												
No/Unknown	1	reference			1	reference						
Yes	1.26	1.08	1.48	0.004	0.94	0.78	1.13	0.515				
Do you think your child is preoccupied with the Internet?												
No/Unknown	1	reference			1	reference						
Yes	1.46	1.33	1.61	< 0.001	1.12	0.98	1.27	0.092				
Do you think your child needs to use the Internet with increasing amounts of time in order to achieve satisfaction?												
No/Unknown	1	reference			1	reference						
Yes	1.4	1.27	1.54	< 0.001	0.98	0.86	1.13	0.816				
Do you think your child repeatedly makes unsuccessful efforts to control, cut back, or stop Internet use?												
No/Unknown	1	reference			1	reference			1	reference		
Yes	1.58	1.42	1.76	< 0.001	1.13	0.98	1.31	0.097	1.15	1	1.32	0.058
Do you think your child feels restless, moody, depressed, or irritable when attempting to cut down or stop Internet use?												
No/Unknown	1	reference			1	reference			1	reference		
Yes	1.74	1.55	1.95	< 0.001	1.43	1.24	1.65	< 0.001	1.48	1.29	1.7	< 0.001
Do you think your child stays on-line longer than originally intended?												
No/Unknown	1	reference			1	reference			1	reference		
Yes	1.54	1.4	1.71	< 0.001	1.24	1.08	1.42	0.002	1.27	1.12	1.44	< 0.001
Do you think your child jeopardized or risked the loss of a significant relationship, educational, or extracurricular activities because of the Internet?												
No/Unknown	1	reference			1	reference						
Yes	1.77	1.29	2.42	< 0.001	1.2	0.86	1.68	0.278				
Do you think your child lied to family members, a therapist, or others to conceal the extent of involvement with the Internet?												
No/Unknown	1	reference			1	reference						
Yes	1.46	1.23	1.72	< 0.001	1.09	0.9	1.31	0.368				
Do you think your child uses the Internet as a way of escaping from problems or relieving a dysphoric mood?												
No/Unknown	1	reference			1	reference			1	reference		
Yes	1.66	1.34	2.08	< 0.001	1.26	0.99	1.6	0.059	1.3	1.03	1.64	0.027

*OR* is an abbreviation for Odds ratio

*CI* is an abbreviation for Confidence interval

## Discussion

We evaluated sleep problems and behaviours related to screen-based media use among primary school children. The major findings were that in the instance of increased cumulative scores of the brief sleep questionnaire were associated not with having a digital device, but with feeling restless, moody, depressed, or irritable when attempting to cut down or stop Internet use; staying on-line longer than originally intended; and using the Internet as a way of escaping from problems or relieving a dysphoric mood. We also found that longer weekday sleep duration was associated with less sleep problems, while longer weekend sleep duration was detrimental.

This study used the brief sleep questionnaire for Japanese children developed by a group at the National Centre of Neurology and Psychiatry in 2017 to assess sleep problems in children [36]. The reliability and validity study conducted by the development group was conducted with primary school students (grades 1–6) and reported a mean cumulative score of 24.2 ± 3.6 (range: 19–38) [36]. Compared to that value, the mean cumulative score in this study was slightly higher at 24.7 ± 3.3. In addition, the percentage of children with a threshold of ≥24 was 59.4% in the present study, compared to 53.5% in that 2017 study [36]. The data used for the development of the brief sleep questionnaire were collected in 2009 [45], and the study area was about as urbanised as the areas covered in the current survey; thus, the difference in percentages may be largely due to changes over time, as well as other factors such as sample size, schools, and measures.

Our study showed that the cumulative score was higher for younger (vs. older) students, meaning that they were more likely to have sleep problems. A previous study using the CSHQ-J showed similar results, with cumulative scores being significantly higher in the lower (first and second graders) than in the higher grades (fifth and sixth graders) [45]. The reason for this was that the scores in lower grades were significantly higher than those of the higher graders on the CSHQ-J sub-items ‘resistance at bedtime’ and ‘sleep anxiety’ [45]. The questionnaire included questions about ‘children resisting bedtimes’, and ‘children being afraid of sleeping in the dark’, which may be the reason for the high scores in the lower grades.

In this study, we used the YDQ, which is a very simple and easy way to assess/classify Internet addiction, with the criterion of answering in the affirmative to five or more items. However, we have some concerns about this criterion, as it might not be very sensitive (or specific) to draw an accurate classification/diagnosis. Beard suggested that to be relatively more certain of an accurate classification/diagnosis, the first five items should be answered as ‘yes’ and at least one of the remaining items should also be answered as ‘yes’ [46]. Further study is needed when using the YDQ in clinical situations.

The proportion of children addicted to the Internet seems to change over time, depending on the age of those surveyed and the timing of the survey. A previous Japanese study in 2019 used the same YDQ as the one used in this study, and reported the percentage of upper grade children who met five or more criteria as 8.4% for boys and 4.2% for girls [47]. A 2018 survey of Japanese junior high school students using Young’s Internet Addiction Test reported that 4.3% were ‘addicted’ and 26.3% were ‘possibly addicted’ [13]. In another study using the YDQ in 2016–17, the percentage of children who met the criterion of five or more items was 17 and 6% for first to third grade boys and girls, respectively, and 12 and 7% for fourth to sixth grade boys and girls, respectively [48]. The percentage of lower grade boys in this study was about the same as the 17% in the previous study; however, the percentage of lower grade girls was higher. As for the percentage of upper grade students, the percentage was 2–3 times higher for both sexes than that in the two previous studies. In a 2008 study in Greece that also used the YDQ, the percentage of adolescents who met the criterion of five or more items was 11% [49].

In this study, associations were observed between the higher cumulative score of the brief sleep questionnaire for Japanese children and sex, grade, weekday sleep duration, and weekend sleep duration. Results indicated that boys had lower scores than girls. As for grades, there was no significant difference in first or second grade, but for third grade and above, the ORs became significantly smaller as the grade increased. It was found that the higher the grade, the lower the score. Moreover, a longer sleep duration on weekdays had a protective effect on the score, while longer sleep duration on weekends was a risk factor for raising the score. Less sleep time on weekdays indicated a later bedtime. Additionally, more sleep on weekends may indicate a situation where individuals may be compensating for the weekday sleep portion (i.e. sleep debt) [50]. This is an important issue in children’s sleep, as evidence supports an association between differences in weekday and weekend sleep and academic performance, depressive symptoms, substance use, and risk of overweight/obesity [51, 52].

We created a model adjusted for sex, grade, weekday sleep duration, weekend sleep duration and a reduced model with backward elimination adjusted for the same variables to examine the association between children with a cumulative score ≥ 24 on the brief sleep questionnaire, the digital devise ownership, and Internet addictive behaviour. Having one’s own digital device was associated with children with a cumulative score ≥ 24 on the brief sleep questionnaire in univariate analyses; however, the association was lost in logistic regression analyses using the backward elimination method. A previous study showed that portable electronic devices are more strongly associated with sleep duration than non-portable electronic devices, and the association is maintained when demographic variables, diagnoses of anxiety and depression, physical activity, and body mass index are included in the model [53]. The results of this study suggest the need to distinguish between sleep duration and problems when considering the association between electronic devices and children’s sleep. In the reduced model, the item ‘have own digital device’ was removed, leaving the following items: ‘feel restless, moody, depressed, or irritable when attempting to cut down or stop Internet use’; ‘stay on-line longer than originally intended’; and ‘use the Internet as a way of escaping from problems or for relieving a dysphoric mood’—all of which had significant effects on children’s cumulative score of the brief sleep questionnaire. These items, especially the association between Internet use to escape a dysphoric mood and the children’s score, may suggest the presence of some problems or anxiety.

Anxiety often leads to sleep problems [54, 55]. Excessive worry and fear can make it difficult to fall asleep and stay asleep throughout the night. Sleep deprivation can worsen anxiety and add to a negative cycle that includes insomnia and anxiety disorders [56]. Stress resulting from any problem also activates the sympathetic nervous system and the hypothalamic-pituitary-adrenal axis, and the activation stimulates the secretion of corticotropin-releasing hormone, which further induces sleep disturbances. Indeed, stress [57] and stressful times can have negative effects on adolescents’ sleep [58]. Mental or psychological problems may also be potentially causing an individual to ‘feel restless, moody, depressed, or irritable when attempting to cut down or stop Internet use’ and ‘stay on-line longer than originally intended’. In adolescents, low self-regulation is independently associated with greater daytime sleepiness and greater eveningness chronotype, but not with shorter sleep duration [59]. A study of children and adolescents with attention-deficit/hyperactivity disorder found that children with higher deficits in emotional self-regulation also had higher sleep problems [60].

We speculate that the link between Internet use and the cumulative score of the brief sleep questionnaire may be owing to children’s emotional and psychological development. A study of primary school students reported that an Internet-dependence tendency was significantly associated with mental health, relationship with parents, communication, normative consciousness, and aggression; and that time spent on the Internet was associated only with mental health when adjusting for grade and sex [61]. It can be difficult for children to cope with emotional and psychological factors, such as anxiety and self-regulation, that can affect their sleep problems. Parents and teachers may, therefore, need to identify problems as early as possible and consult health professionals [62]. Children’s screen media use and sleep could benefit from a public health approach [63, 64]. In the future, it is important to raise awareness about this among children, parents, and teachers [57, 65].

This study had several limitations. First, since the research design was cross-sectional, causal relationships could not be ascertained. In the future, longitudinal or intervention studies are needed to closely examine the effects of digital device use on children’s sleep. Second, since the YDQ was answered by parents/guardians, the responses related to objective facts were highly reliable; however, those related to subjectivity may not be as reliable. Therefore, it is necessary to conduct a survey to confirm the validity of the responses when the YDQ is answered by parents. Third, the study used the brief sleep questionnaire for children in Japan, (a shortened version of the CSHQ-J which has been validated in translation with Owens, the developer of the original CSHQ), but its validation has not been completed yet. Finally, the lack of assessment of physical activity levels, sociodemographic level of parents/guardians, and other important potential covariates does not remove the possibility of these effects.

## Conclusion

The results suggest that the relationship between screen-based media use and children’s sleep problem is not simply that media use leads to less sleep, but that mental or psychological factors may contribute to the use of media by children with sleep problems. Parents and teachers may need to address screen-related sleep problems in children, as they may be influenced by mental or psychological factors. It can be foreseen that screen use will become even more present in children’s lives. Further research is also needed to emphasise the relationship between screen-based media use and children’s sleep, as sleep is an important factor in child development.

## Data Availability

The datasets used and/or analysed on current study are available from the corresponding author on reasonable request.

## Abbreviations

CI: Confidence interval
ICT: Information and communication technology
CSHQ: Children’s Sleep Habits Questionnaire
CSHQ-J: Japanese version of the Children’s Sleep Habits Questionnaire
YDQ: The Young Diagnostic Questionnaire for Internet Addiction
OR: Odds ratio
